# Incidence and Timing of Foot Injury Diagnosis in Polytrauma Patients: A Retrospective Registry-Based Study

**DOI:** 10.1177/24730114251398763

**Published:** 2025-12-23

**Authors:** Aleksi Säkkinen, Antti Riuttanen, Ville Mattila, Heikki Mäenpää, Nikke Partio

**Affiliations:** 1 Tampere University Hospital, Tampere, Finland; 2 Tampere University Faculty of Medicine and Health Technology, Tampere, Finland; 3 Coxa Hospital Ltd, Tampere, Finland

**Keywords:** polytrauma, foot fractures, delayed diagnosis, Lisfranc injury, Chopart injury, Hawkins classification, Sanders classification

## Abstract

**Background:**

Foot injuries are often missed in polytrauma patients, as more severe injuries may overshadow them, leading to delayed diagnosis and treatment. The aim of this study was to assess the incidence of foot injuries in polytrauma patients, evaluate the timing of their diagnosis, and identify risk factors associated with delayed diagnosis.

**Methods:**

In this retrospective registry-based study, all polytrauma patients with a New Injury Severity Score of ≥16 treated at Tampere University Hospital (TAUH) from 2016 to 2023 were screened for foot fractures as well as Lisfranc and Chopart injuries. Patient demographics, injury characteristics, and timing of diagnosis were extracted from TAUH’s trauma registry and analyzed.

**Results:**

Out of 1327 polytrauma patients, 54 (4.1%) sustained foot injuries, totalling 215 foot injuries (195 fractures). Delayed diagnosis, defined as >24 hours between trauma and diagnosis, occurred in 23 patients (43%), involving 80 injuries (37%). Fractures of the midfoot and metatarsus were most commonly diagnosed with delay. Delayed diagnosis was significantly more common in patients with a higher number of foot fractures and injuries (*P* < .001). Logistic regression analysis identified number of foot injuries (OR 1.658 [95% CI 1.098-2.504 ]), lower Glasgow Coma Scale (GCS) scores (OR 0.582 [95% CI 0.340-0.998]), and presence of concomitant facial injuries (OR 18.227 [95% CI 1.643-202.211]) as independent risk factors for delay in diagnosis.

**Conclusion:**

A substantial portion of polytrauma patients had foot injuries that were diagnosed >24 hours after the time of trauma. Despite these delays, most injuries were minor and without notable clinical consequences. Current tertiary survey protocols effectively detect most major foot injuries requiring immediate attention.

**Level of Evidence:**

Level III, retrospective registry.

## Introduction

In earlier studies it has been reported that 5.0% to 7.3% of polytrauma patients sustained fractures of the foot, with metatarsal fractures being the most common.^1,2^ Advancements made in first-aid, intensive care and operating techniques have significantly reduced mortality in polytrauma patients, increasing the importance of quality of life after trauma.^3^ Although secondary to major injuries, foot injuries can have a significant impact on post-trauma quality of life.^4,5^

Non-life-threatening extremity injuries may be initially neglected because of prioritization of more severe injuries.^6^ A previous study reported that the diagnosis of foot injuries in polytrauma patients was delayed in 33% of cases, defined as >24 hours after trauma.^1^ A review on delayed diagnosis of trauma injuries reported that 8.1% to 25.8% of patients experienced delayed diagnosis of foot or ankle injuries, defined as any injury identified after the primary assessment or after admission to intensive care.^7^ Some studies included patients with an Injury Severity Score (ISS) lower than 16, limiting comparability because higher ISS is linked to delayed diagnosis.^8^ Risk factors for delayed diagnosis of foot injuries reported in earlier studies are a high ISS score, severe traumatic brain injury, admission to the intensive care unit (ICU), and advanced age.^1,2,7,8^

Delayed diagnosis of foot injuries reportedly does not significantly impact treatment outcomes in polytrauma patients.^1,2^ van der Vliet et al^1^ found that only 8% of foot fractures with a delay in diagnosis required operative treatment. Ahrberg et al^2^ reported that 10% of polytrauma patients could have benefitted from earlier diagnosis of foot injury.

The aims of this study were to determine the incidence and distribution of foot fractures and Lisfranc and Chopart injuries. Secondarily, we examined the timing of diagnosis and assessed possible risk factors for delayed diagnosis.

## Methods

### Study Design and Patients

This retrospective study used data from the Tampere University Hospital (TAUH) trauma registry. All registry patients are severely injured, fulfilling the New Injury Severity Score (NISS) ≥16 criterion, and have been treated in the ICU. TAUH serves as the tertiary trauma center for the surrounding 3 hospital districts, providing comprehensive trauma care across all medical specialties. TAUH covers a population base of approximately 900 000 people and is responsible for the most complex trauma cases in its catchment area.^9^ Patients of all ages were eligible for this study. Polytrauma patients were screened for foot injuries using diagnostic codes.

Polytrauma patients (NISS ≥16) with foot injuries treated at TAUH between 2016 and 2023 were included in this study.

### Outcome Measures and Explanatory Variables

A commonly used method for classifying the severity of injuries is the Abbreviated Injury Scale (AIS). The AIS divides the body into 9 regions and assigns a severity score to each injury on a scale from 1 (minor) to 6 (fatal).

To assess overall injury severity, 2 scoring systems are often used: the ISS and the NISS.

- ISS: calculated by summing the squares of the AIS scores of the 3 most severe injuries *from different body regions*
- NISS: calculated by summing the squares of the AIS scores of the 3 most severe injuries *regardless of body region*

A patient was classified as a polytrauma patient if they had a NISS of ≥16.

For all patients who fit the criteria mentioned above, data on age, sex, date of admission, length of hospital stay, length of ICU admission, NISS, ISS, Glasgow Coma Scale (GCS) score, and mechanism of injury were extracted from the registry. Fractures of the calcaneus, talus, naviculare, cuboideum, cuneiform, metatarsus, sesamoideum and phalanges, as well as Lisfranc and Chopart injuries were recorded. Lisfranc and Chopart injuries were recorded separately from other foot fractures, as they represent complex midfoot joint injuries that may be purely ligamentous or associated with fractures. For this study, both the ligamentous injury and any associated fractures were documented, with fractures counted in the respective anatomical categories. All fractures were defined as either open or closed. Treatment of injuries was defined as either operative or conservative. Operative treatment was defined as any surgical intervention that directly addresses the fracture or injury, involving surgical techniques aimed at stabilizing or repairing the bone. Purely soft tissue procedures, such as wound closure, did not qualify as operative treatment.

Intra-articular calcaneal and talar neck fractures were further classified by their severity using Sanders and Hawkins classification, respectively.^10-14^ The Sanders classification for calcaneal fractures is based on the number of articular fractures as described by Sanders et al.^14^ The Hawkins classification is based on the degree of displacement and dislocation as described by Hawkins.^12^ Both systems classify the severity of a fracture on a scale of I-IV and aim to serve as a guide for treatment selection and prediction of clinical outcome.

Lisfranc and Chopart injuries are complex injuries of the midfoot, and they often require operative treatment because of instability and risk of long-term functional impairment.^15,16^ Both injuries can occur in isolation as purely ligamentous disruptions, but they are frequently associated with fractures of the surrounding bones.^15,17^

Injuries were divided into groups based on timing of diagnosis: acute (diagnosed within 24 hours) and delayed (diagnosed after 24 hours). Injuries diagnosed more than 2 weeks after trauma were considered a subgroup of delayed injuries and defined as missed injuries.

### Statistical Analysis

Statistical analysis was performed with SPSS version 29.0.2.0 for Windows. Data were reported as medians noted with interquartile range. To compare explanatory variables between groups, χ^2^ and Mann-Whitney *U* tests were used. The χ^2^ test was used for categorical variables and the Mann-Whitney *U* test was used for continuous variables, as all continuous variables were non-normally distributed. The Fisher exact test was used for categorical variables when the expected cell count was ≤5.^18^

A logistic regression model was used to account for confounding factors and analyse independent association with delay in diagnosis of foot injury. For the purpose of logistic regression, patients were divided into 2 groups based on the timing of diagnosis of their foot injuries. One group included all patients with all foot injuries diagnosed less than 24 hours after trauma, whereas the other group included all patients with foot injuries diagnosed more than 24 hours after trauma. Patients with missing data were excluded from the logistic regression analysis. The following covariates were included in the multivariable logistic regression model: age, sex, NISS, GCS, length of ICU stay, length of hospital stay, number of foot injuries, and the presence of concomitant head, facial, chest, abdominal and external injuries.

A *P* value of ≤.05 was considered to be statistically significant.

## Results

Between 2016 and 2023, a total of 1327 polytrauma patients (NISS ≥16) were treated at TAUH. Using diagnostic codes, we identified 54 patients (4.1%) with 1 or more foot injuries. All patients with Lisfranc or Chopart injuries had concomitant foot fractures.

The median age of polytrauma patients with foot injuries was 37. A majority of these patients, 41 in total, were men (76%). The most common mechanism of injury was falling from height, with 22 patients sustaining this mechanism of trauma. Of these patients, all but 1 fell from a height greater than 3 m. The second most common mechanism of injury were traffic accidents. Median GCS at admission was 14. GCS at admission was missing from 3 patients. The median NISS was 27 and the median ISS was 21. Median duration of stay in the hospital was 15 days. Median duration of stay in the ICU was 2 days. Length of stay in the ICU was missing for 1 patient. A majority of patients had concomitant thoracic or abdominal injuries (Table 1).

**Table 1.** Baseline Patient Characteristics (N = 54).

Characteristic	Median (IQR)
Age at trauma, y	37 (26-56)
GCS score at presentation^a^	14 (12-15)
NISS	27 (19-33)
ISS	21 (16-28)
Hospital stay, d	15 (6-24)
ICU stay^b^, d	2 (1-7)
	n (%)^c^
Male gender	41 (76)
ICU admission	53 (98)
Concomitant head injury	25 (46)
Concomitant face injury	13 (24)
Concomitant thoracic injury	33 (61)
Concomitant abdominal injury	33 (61)
Concomitant extremities injury	54 (100)
Concomitant external injury	19 (35)
Mechanism of injury	
Fall	22 (41)
>3 m	21 (39)
<3 m	1 (2)
Car accident	21 (41)
Motorcycle crash	7 (13)
Pedestrian	3 (6)
Other	1 (2)

Abbreviations: GCS, Glasgow Coma Scale; ICU, intensive care unit; ISS, Injury Severity Score; NISS, New Injury Severity Score.

a GCS score at presentation was available for 51 of 54 patients (94%).

b Length of ICU stay data were available for 53 of 54 patients (98%).

c Percentages were calculated from total patient count (N = 54).

In total, 195 foot fractures, 5 Chopart, and 15 Lisfranc injuries were reported, totalling 215 injuries of the feet. The most common foot injuries were midfoot and metatarsal fractures with a total of 63 of each being recorded. Thirty-six calcaneal fractures were reported, making it the most often fractured single bone. Of 215 injuries, 46 (21%) were open fractures. Calcaneal and talar fractures were the most common to be open fractures. A majority of injuries were treated conservatively, with 65 (30%) injuries requiring operative treatment. Calcaneal injuries were the most common to require operative treatment, with 21 of 36 (58%) calcaneal fractures requiring operative treatment. On the other hand, navicular fractures were the injury most likely to be treated conservatively, with only 2 of 13 fractures (15%) requiring operative treatment (Table 2).

**Table 2.** Foot Injury Distribution With Open Fracture Rates and Surgical Management (N = 54).^a^

Foot Fractures	n (%)	Open Fracture (%)	Surgery (%)
Calcaneal	36 (18)	15 (42)	21 (58)
Talar	19 (10)	7 (37)	10 (53)
Midfoot	63 (32)	10 (16)	13 (21)
Cuneiform	35 (18)	4 (11)	9 (26)
Cuboid	15 (8)	2 (13)	2 (13)
Navicular	13 (7)	4 (31)	2 (15)
Metatarsal	63 (32)	6 (10)	13 (21)
I	10 (5)	0 (0)	3 (30)
II	15 (8)	2 (13)	3 (20)
III	15 (8)	2 (13)	4 (27)
IV	14 (7)	2 (14)	2 (14)
V	9 (5)	0 (0)	1 (11)
Phalangeal	13 (7)	5 (38)	3 (23)
Sesamoidal	1 (1)	0 (0)	0 (0)
Total foot fractures	195	43 (22)	60 (31)
Ligamentous injuries			
Lisfranc	15 (7)	2 (13)	3 (20)
Chopart	5 (2)	1 (20)	2 (40)
Total foot injuries	215	46 (21)	65 (30)

a Percentages for foot fractures were calculated from total foot fracture count (195), and percentages for Lisfranc and Chopart injuries were calculated from total foot injury count (215).

Of 215 injuries, a total of 80 (37%) were diagnosed more than 24 hours after time of trauma. Seventy-two foot fractures (37%), 6 Lisfranc (40%), and 2 Chopart injuries (40%) were diagnosed in this manner. Notably, 2 injuries were diagnosed more than 14 days after the time of trauma. Both of these were injuries of the midfoot, 1 navicular and 1 cuneiform medialis fracture. Fractures of the midfoot, metatarsus, and phalanges were most commonly diagnosed with delay. On the contrary, most calcaneal and talar fractures were diagnosed less than 24 hours after the time of trauma, with only 3 calcaneal (8%) and 5 talar (26%) fractures being diagnosed more than 24 hours after trauma (Table 3).

**Table 3.** Foot Fractures and Ligament Injuries by Timing of Diagnosis (N = 54).^a^

Foot Fractures	Total	Acute, n (%)	Delayed, n (%)	Missed, n (%)
Calcaneal	36	33 (92)	3 (8)	0 (0)
Talar	19	14 (74)	5 (26)	0 (0)
Midfoot	63	34 (54)	29 (46)	2 (3)
Cuneiform	35	19 (54)	16 (46)	1 (3)
Cuboid	15	8 (53)	7 (47)	0 (0)
Navicular	13	7 (54)	6 (46)	1 (8)
Metatarsal	63	35 (56)	28 (44)	0 (0)
I	10	6 (60)	4 (40)	0 (0)
II	15	8 (53)	7 (47)	0 (0)
III	15	8 (53)	7 (47)	0 (0)
IV	14	7 (50)	7 (50)	0 (0)
V	9	6 (67)	3 (33)	0 (0)
Phalangeal	13	7 (54)	6 (46)	0 (0)
Sesamoidal	1	0 (0)	1 (100)	0 (0)
Total foot fractures	195	123 (63)	72 (37)	2 (1)
Ligamentous injuries				
Lisfranc	15	9 (60)	6 (40)	0 (0)
Chopart	5	3 (60)	2 (40)	0 (0)
Total foot injuries	215	135 (63)	80 (37)	2 (1)

a Percentages were calculated based on the total number of cases for each specific injury type.

Acute: diagnosed within 24 hours; Delayed: diagnosed after 24 hours; Missed: diagnosed after 14 days.

Intra-articular calcaneal fractures and talar neck fractures were classified according to Sanders and Hawkins classification systems, respectively. Of 36 calcaneal fractures, 29 (81%) were intra-articular. A majority of these fractures, 22 of 36 (61%), were classified as type IV injuries. The rest were evenly distributed across the other types with 3 (8%) type I, 2 (6%) type II, and 2 (6%) type III intra-articular calcaneal fractures. Of 19 talar fractures, 16 (84%) were talar neck fractures. Talar neck fractures were more evenly distributed among the classifications scale with 8 (42%) type I, 1 (5%) type II, 4 (21%) type III, and 3 (16%) type IV talar neck fractures.

A total of 31 (57%) patients had all foot injuries diagnosed in <24 hours after trauma. A total of 23 (43%) patients had at least 1 foot injury that was diagnosed more than 24 hours after trauma. No patient had an isolated delay in diagnosis of a Lisfranc or Chopart injury. No significant differences between the 2 groups were reported in most baseline characteristics. Patients with at least 1 foot injury diagnosed more than 24 hours after trauma had a significantly higher number of foot fractures and injuries (*P* < .001) (Table 4).

**Table 4.** Baseline Characteristics of Patients by Timing of Diagnosis (N = 54).

	Acute (n = 31), Median (IQR)	Delayed (n = 23), Median (IQR)	*P* Value^a^
Age at trauma, y	38 (28-55)	36 (19-62)	.649
GCS score at presentation^b^	15 (14-15)	14 (5-15)	.071
NISS	27 (22-34)	27 (17-33)	.441
ISS	22 (14-29)	20 (17-24)	.806
Hospital stay, d	16 (6-24)	11 (6-27)	.986
ICU stay^c^, d	3 (1-6)	2 (1-8)	.670
Number of foot fractures	1 (1-3)	4 (2-7)	**.001**
Number of foot injuries	2 (1-4)	5 (3-8)	**.001**
	n (%)	n (%)	
Male gender	24 (77)	17 (74)	.766
Concomitant head injury	13 (42)	12 (52)	.456
Concomitant face injury	4 (13)	9 (39)	.051^d^
Concomitant thoracic injury	20 (65)	13 (57)	.551
Concomitant abdominal injury	22 (71)	11 (48)	.085
Concomitant external injury	10 (32)	9 (39)	.601

Abbreviations: GCS, Glasgow Coma Scale; ICU, intensive care unit; ISS, Injury Severity Score; NISS, New Injury Severity Score.

a Boldface indicates significance (*P* < .05).

b GCS score at presentation was available for 51 of 54 patients (94%).

c Length of ICU stay data were available for 53 of 54 patients (98%).

d *P* value for concomitant face injury calculated with Fisher exact test as expected cell count was <5.

Logistic regression identified number of foot injuries (OR 1.658 [95% CI 1.098-2.504 ], *P* = .016), lower GCS scores (OR 0.582 [95% CI 0.340-0.998], *P* = .049), and presence of concomitant facial injuries (OR 18.227 [95% CI 1.643-202.211], *P* = .018) as independent risk factors for delay in diagnosis.

Four patients were excluded from the analysis because of missing data. Three patients were missing GCS score at admission and 1 was missing length of stay data in the ICU. Two of these patients were in the group with no delay in diagnosis, both with missing GCS score at admission, and 2 were in the group with delay in diagnosis, 1 with missing GCS score at admission, and 1 with missing length of ICU stay data.

## Discussion

This study found that foot injuries occurred in 4.1% of polytrauma patients at our institution, with delayed diagnosis occurring in 43% of affected patients, representing 37% of all foot injuries. The independent risk factors associated with delayed diagnosis were higher number of foot injuries, lower GCS scores, and concomitant facial injuries.

An important finding of our study was the identification of independent risk factors associated with delayed diagnosis of foot injuries in polytrauma patients. Multiple foot injuries may complicate clinical assessment because of overlapping symptoms, leading to delayed recognition of individual injuries during initial trauma evaluation. A reduced level of consciousness, reflected by lower GCS, limits the patient’s ability to report pain and may result in injuries of the foot being deprioritized during primary and secondary surveys. In addition, patients with lower GCS score commonly have severe injuries of the head that require immediate attention, diverting focus from injuries of the extremities. Despite this, the presence of head injuries, which have a strong correlation with a reduced level of consciousness, were not independently associated with delay in diagnosis. The association between facial injuries and delayed diagnosis of foot injuries likely reflects practical challenges in the acute setting; patients with severe craniofacial trauma are often intubated or unable to communicate, limiting the assessment of foot pain and thorough examination. This association may further be explained by high-energy trauma mechanisms, which predispose patients to both craniofacial injuries and complex foot fractures. These findings differ from existing literature, as in a similar study only age was identified as an independent risk factor for delay in diagnosis.^1^

Our findings align with existing literature regarding the incidence of foot injuries in polytrauma patients. The observed rate of 4.1% falls slightly lower than the range reported by previous studies, which found incidences between 5.0% and 7.3%.^1,2^ However, our study differs in the distribution of injury types, with midfoot fractures being most common, closely followed by metatarsal fractures. This finding differs with earlier studies that predominantly report metatarsal fractures as most frequent.^1,2,7,19^ The higher prevalence of falls from height as the mechanism of injury in our cohort (41% vs lower rates in previous studies) may explain this discrepancy, as falls from significant heights typically result in high-energy axial loading that commonly affects the hindfoot and midfoot structures. The rate of operative intervention (30%) and open fractures (21%) in our series was consistent with previous reports, reinforcing the validity of our findings.^1,2^ We also found that calcaneal and talar fractures were the most likely to require operative treatment, which aligns with earlier findings, with 58% of calcaneal fractures and 47% of talar fractures requiring surgical intervention.

We found that 37% of foot fractures, affecting 43% of patients, were diagnosed with delay. This rate is somewhat higher than that reported by van der Vliet et al,^1^ who found delayed diagnosis in 30% of fractures and 33% of patients. Several factors may contribute to this difference. The complexity of injuries in our patient population, evidenced by higher median NISS scores, may have increased the likelihood of overlooking secondary injuries during initial assessment. A notable finding was the pattern of delay in diagnosis based on injury location. Fractures of the midfoot, metatarsus, and phalanges were most commonly diagnosed with delay, whereas calcaneal and talar fractures were typically identified within 24 hours. Only 3 (8%) calcaneal and 5 (26%) talar fractures were diagnosed more than 24 hours after trauma, compared with significantly higher rates for other locations. This pattern suggests that larger, more obvious fractures with significant clinical signs are easily identified during initial assessment, whereas smaller bone injuries may be overlooked because of subtle presentation of symptoms. The same pattern has also been reported in existing literature.^1,2^

Only 1 injury, which was diagnosed with delay, was treated operatively. This patient could have benefitted from an earlier diagnosis, avoiding the additional anaesthesia required for a second surgery. The remaining injuries that were diagnosed with delay were generally minor, which is reflected in the absence of surgical intervention. This suggests that, although delayed diagnosis occurred in several cases, the clinical impact was limited in most patients. These factors indicate that current tertiary survey protocols effectively detect major foot injuries requiring immediate attention.

Lisfranc and Chopart injuries are rare, with widely varying reported incidence rates. Lisfranc injuries account for approximately 0.2% of all fractures with an incidence of 1.8-11.4 per 100 000 person-years,^17,20,21^ whereas Chopart injuries are even less common with an estimated incidence of around 2.2-4 per 100 000 person-years.^21,22^ Studies have reported that up to 20% of Lisfranc injuries are initially missed during the acute evaluation of trauma patients,^23^ and the rate of missed Chopart injuries has been estimated to be up to 41%.^24^ Missed or delayed diagnosis can lead to persistent pain, instability, post-traumatic arthritis, and poor functional outcomes.^24-26^

The incidence of Lisfranc injuries was 1.1% and Chopart injuries was 0.4% for polytrauma patients in this study. The incidence of Chopart and Lisfranc fractures in polytrauma patients has not been assessed in earlier studies, which makes this study a novel contribution to the understanding of these types of foot injuries in severely injured patients. The rate of delayed diagnosis for both Chopart and Lisfranc injuries was 40%, which is similar to previously reported rates for delayed diagnosis in these injury types.^23,24^

This study has several limitations. First, this was a retrospective registry-based study; therefore, we were limited by the availability of data as well as their accuracy. Second, our sample size was relatively small, as only 54 polytrauma patients with foot injuries were included of the initial 1327 patients screened, which undermines the statistical significance of our findings. The single-center design of this study limits generalizability to other trauma systems with different protocols and resources.

The strengths of this study include the use of a prospectively collected cohort of severely injured patients, which closely reflected the trauma population typically treated at a tertiary care center. In addition, access to comprehensive clinical data, including all radiologic images, reports, and patient records, enabled extensive verification and classification of foot injuries, which is not always feasible in purely registry-based studies.

## Conclusion

Foot injuries are an uncommon occurrence in polytrauma patients. Although a substantial number of foot injuries were diagnosed with delay, most were minor and did not require operative intervention, indicating that the overall clinical impact of delayed diagnosis was limited in our cohort. Nevertheless, tertiary survey should be an ongoing process to prevent delayed diagnosis of foot injuries. A large portion of foot injuries go unnoticed in the primary and secondary survey that may have clinical consequences. Based on our findings, we recommend implementation of structured tertiary survey protocols with mandatory foot examination components, particularly for high-risk patients. When one foot injury is identified, systematic examination of the entire foot and contralateral extremity should be compulsory, given the high frequency of multiple foot injuries observed. In addition, patients presenting with a low GCS score on admission or concomitant facial injuries should receive targeted evaluation for foot injuries, as this subgroup was associated with a higher risk of delayed diagnosis in our cohort.

## Supplemental Material

sj-pdf-1-fao-10.1177_24730114251398763 – Supplemental material for Incidence and Timing of Foot Injury Diagnosis in Polytrauma Patients: A Retrospective Registry-Based Study Supplemental material, sj-pdf-1-fao-10.1177_24730114251398763 for Incidence and Timing of Foot Injury Diagnosis in Polytrauma Patients: A Retrospective Registry-Based Study by Aleksi Säkkinen, Antti Riuttanen, Ville Mattila, Heikki Mäenpää and Nikke Partio in Foot & Ankle Orthopaedics
